# Accessing the genomic information of unculturable oceanic picoeukaryotes by combining multiple single cells

**DOI:** 10.1038/srep41498

**Published:** 2017-01-27

**Authors:** Jean-François Mangot, Ramiro Logares, Pablo Sánchez, Fran Latorre, Yoann Seeleuthner, Samuel Mondy, Michael E. Sieracki, Olivier Jaillon, Patrick Wincker, Colomban de Vargas, Ramon Massana

**Affiliations:** 1 Department of Marine Biology and Oceanography, Institute of Marine Sciences (ICM)–CSIC, Pg. Marítim de la Barceloneta, 37-49, Barcelona E-08003, Spain; 2 CEA, Institut de Génomique, Génoscope, 2 Rue Gaston Crémieux, Evry F-91000, France; 3 CNRS, UMR 8030, CP5706, Evry, F-91000, France; 4 Université d’Evry, UMR 8030, CP5706, Evry, F-91000, France; 5 National Science Foundation, 4201 Wilson Boulevard, Arlington, VA 22230, USA; 6 Bigelow Laboratory for Ocean Sciences, 60 Bigelow Drive, East Boothbay, ME 04544, USA; 7 CNRS, UMR 7144, Station Biologique de Roscoff, Place Georges Teissier, Roscoff, F-29680, France; 8 Sorbonne Universités, UPMC Université Paris 06, UMR 7144, Station Biologique de Roscoff, Place Georges Teissier, Roscoff, F-29680, France

## Abstract

Pico-sized eukaryotes play key roles in the functioning of marine ecosystems, but we still have a limited knowledge on their ecology and evolution. The MAST-4 lineage is of particular interest, since it is widespread in surface oceans, presents ecotypic differentiation and has defied culturing efforts so far. Single cell genomics (SCG) are promising tools to retrieve genomic information from these uncultured organisms. However, SCG are based on whole genome amplification, which normally introduces amplification biases that limit the amount of genomic data retrieved from a single cell. Here, we increase the recovery of genomic information from two MAST-4 lineages by co-assembling short reads from multiple Single Amplified Genomes (SAGs) belonging to evolutionary closely related cells. We found that complementary genomic information is retrieved from different SAGs, generating co-assembly that features >74% of genome recovery, against about 20% when assembled individually. Even though this approach is not aimed at generating high-quality draft genomes, it allows accessing to the genomic information of microbes that would otherwise remain unreachable. Since most of the picoeukaryotes still remain uncultured, our work serves as a proof-of-concept that can be applied to other taxa in order to extract genomic data and address new ecological and evolutionary questions.

Most marine biodiversity is constituted by microbes that dominate in biomass and are fundamental for ecosystem functioning and biogeochemical processes^1^,^2^,^3^. Among them, microbial eukaryotes play significant roles in primary production^4^, nutrient cycling, and food-web dynamics as grazers and parasites^5^,^6^. In particular, pico- and nano-sized Heterotrophic Flagellates (HF; 1–5 μm) are important mortality agents of planktonic prokaryotes. Furthermore, HF constitute a key link in the transfer of organic carbon to upper trophic levels^7^. For a long time, marine HF were studied as homogeneous assemblages, but molecular surveys have revealed that they include evolutionary very diverse groups^6^. A notable component of HF assemblages are the MArine STramenopiles or MASTs, which are constituted by at least 18 groups^8^ with widespread distributions^9^,^10^,^11^,^12^. MASTs may reach up to 35% of cells in the HF assemblage^13^ and one group in particular, the geographically widespread MAST-4, can present cell abundances averaging 9% of HF^13^ in the marine euphotic layer. Thus, MAST-4 may be one of the most abundant HF in the oceans. Unfortunately, MAST species have escaped cultivation so far, with only one exception within the clade MAST-3^14^. Despite the obvious importance of MAST cells, both in terms of abundance and diversity, little is known about their biology and evolution. To address the latter, genome sequencing appears as a powerful approach, but the lack of sufficient genomic DNA material due to cells’ unculturability prevents traditional shotgun sequencing and genome assembly.

Currently, there still is a big knowledge gap in protist genomics caused by the reluctance of many species to grow in culture. Indeed, only 15% of the completed or ongoing projects in the Genomes OnLine Database (GOLD; https://gold.jgi.doe.gov)^15^ concern protistan taxa, and most of them come from cultured phototrophic^16^,^17^,^18^ or parasitic species^19^, resulting in a biased view of the full eukaryotic diversity^20^. In this context, a natural option is single cell genomics (SCG), which produce Single Amplified Genomes (SAGs) that can later be sequenced. This technology was initially used to produce genomic information from single prokaryotic cells collected from the ocean^21^. The first SCG studies targeting microeukaryotes aimed at getting an accurate assessment of community composition^22^, or exploring complex biotic interactions at the single-cell level, such as the presence of prey or pathogens within a host cell^23^,^24^. To the best of our knowledge, only three studies have applied SCG to retrieve the genomes of uncultured microeukaryotes including Picozoa (3 SAGs)^23^, *Paulinella ovalis* (6 SAGs)^25^ and MAST-4 (1 SAG)^26^. Whereas the genome completeness in SCG studies of prokaryotes range from 10% to 100%^21^, the genomes obtained in the previous eukaryotic studies are still partial. For instance, Roy and colleagues^26^ retrieved about one third of the conserved eukaryotic protein coding genes, used as proxy for genome completeness, in their MAST-4 SAG assembly. This limited recovery is likely produced by the bias introduced during the whole-genome amplification, which seems to preferentially amplify certain genomic regions^27^. A recent study has revealed an average genome recovery per SAG of 81% when compared against the 10.4 Mbp reference genome of *Cryptosporidium parvum*^28^, a pathogenic protist infecting both humans and animals^29^. Unfortunately, most protist species have larger and more complex genomes, lacking also reference genomes that can help during assembly.

Here, we increase the recovery of genomic information from two marine HF species by co-assembling separately-sequenced SAGs belonging to the same species (Supplementary Fig. S1). Different cells from two MAST-4 lineages (clades A and E) were isolated during the *Tara Oceans* expedition^30^ and SAGs were produced. Before co-assembling different SAGs, we ensured that they had identical 18S rDNA, shared comparable tetranucleotide frequencies, and had >95% overall nucleotide identity. We observed a significant increase in the genome recovery in both MAST-4 clades, from around 20% in individual SAGs to 68–74% in the co-assemblies. Our approach allowed recovering genomic functions from genomes that were previously unknown, and which will be pivotal to understand the ecological role of these uncultured flagellates in the ocean.

## Results and Discussion

### Limitations of using only one SAG to investigate genomics of marine picoeukaryotes

A total of 22 pico-heterotrophic cells affiliating to MAST-4A (n = 13) and MAST-4E (n = 9) were isolated during the *Tara Oceans* expedition. All MAST-4E cells and most MAST-4A cells were isolated from the same station in the Mediterranean Sea (station 23, Adriatic Sea), whereas two additional MAST-4A cells derived from the Indian Ocean (station 41, Arabic Sea) (Supplementary Tables S1 and S2). A first Sanger sequencing of the 18S rDNA gene from the MDA product revealed identical sequences among all MAST-4E cells, as well as among most MAST-4A cells (only 2 of the 13 cells had only 1 mismatch with the rest). Overall, 23 SAGs were sequenced (one SAG was sequenced twice) producing on average 24.2 million paired-end Illumina HiSeq 2000 reads per SAG (Supplementary Table S2). The sequencing depth of individual SAGs was similar, with a mean value of 4.9 (±1.4) Gbp (Supplementary Table S2).

**Figure 1:**
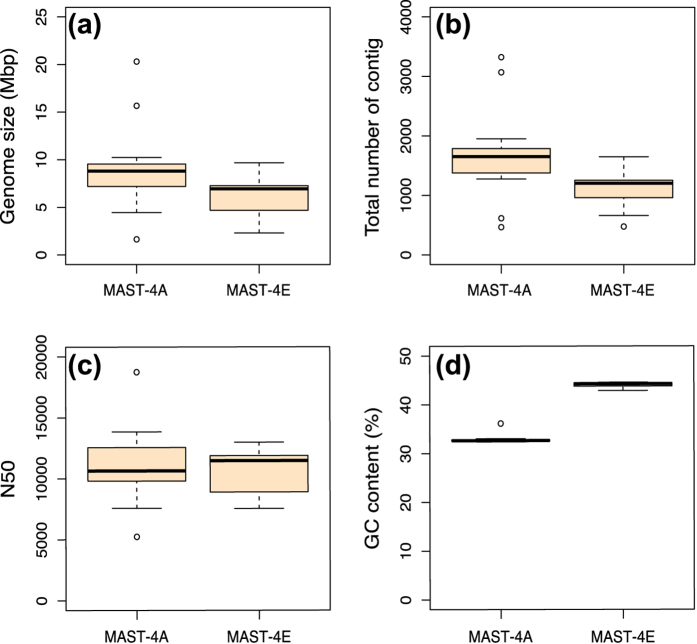
General characteristics of the draft genomes obtained by individual SAGs. Box plots capture the variation in assembly size (**a**), number of contigs (**b**), N50 (**c**) and GC content (**d**) among MAST-4A (n = 14) and MAST-4E (n = 9) SAGs.

Sequenced SAGs were individually assembled, resulting in assembly sizes from 1.6 to 20.3 Mbp in MAST-4A (mean of 9.2 ± 4.5) and from 2.3 to 9.7 Mbp in MAST-4E (mean of 6.2 ± 2.4) (considering contigs >1 kbp; Fig. 1a). This is in agreement with a previous study of one SAG from the MAST-4D lineage that resulted in an assembly size of 16.9 Mbp using a sequencing depth of 6.6 Gbp^26^. Assembly sizes in the other uncultured protists SAGs were similar as well, around 5 Mbp for Picozoa^23^ and *Paulinella ovalis*^25^. Based on these three studies and our own data, it appears that the assembly size obtained by using one SAG may vary by a factor of 10, typically between 2 and 20 Mbp. Similarly, the number of contigs assembled (Fig. 1b) and their respective N50 (Fig. 1c) also varied among SAGs. Within MAST-4A, the contig number varied from 467 to 3,323 (mean of 1,696 ± 764), and the N50 from 5.2 to 18.7 kbp (mean of 11 ± 3.1), while for MAST-4E the contig number varied from 478 to 1,649 (mean of 1,099 ± 351), and N50 from 7.5 to 13.0 kbp (mean of 10.6 ± 1.9). Again, both parameters were similar to the values found in MAST-4D^26^. The GC content averaged 33.9% in MAST-4A SAGs and 44.1% in MAST-4E SAGs (Fig. 1d) and showed very little variability (1 and 0.5%, respectively). Such differences in GC content suggest that MAST-4A and MAST4-E are evolutionary divergent. One MAST-4A SAG had a slightly higher GC content (Fig. 1d), which could be due to (i) “non-targeted” DNA found inside the cell due to infection, prey capture, or symbiosis, (ii) externally associated as attached cells or free DNA, or (iii) contamination during the cell sorting or sequencing. So, a substantial part of this foreign DNA could highlight true organismal interactions^23^, and we made the choice to leave it in our analyses for a possible further exploitation.

**Figure 2:**
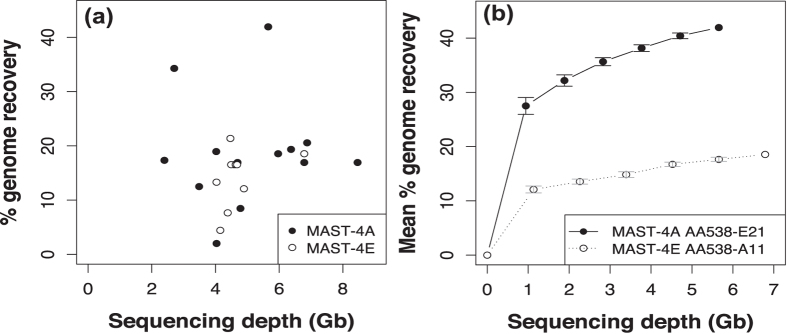
Genome recovery estimated by CEGMA of SAGs in relation to the sequencing effort. (**a**) Genome recovery of the 23 SAGs in relation to their sequencing depth. (**b**) Genome recovery at different sequencing depths in two selected SAGs (those with the largest genome in each clade). Each point represents the mean recovery after 5 separate subsamplings (at 17%, 33%, 50%, 67%, and 83%) of the total number of reads.

The variability in assembly size did not depend on sequencing depth, as shown by the comparison of estimated genome recovery (as percentage of ultra-conserved eukaryotic genes retrieved with CEGMA) *vs*. sequencing depth (Fig. 2a). Genome recovery averaged 18.7% (±9.7) in MAST-4A and 14.1% (±5.4) in MAST-4E SAGs. In some SAGs, genome recovery was similar to the 37.5% that we estimated for the previously sequenced MAST-4D^26^. Furthermore, SAGs that were independently sequenced in two sequencing centers (AA538-G20 and AA538-G20_bis, Supplementary Table S2) produced a similar assembly size (9.2 and 10.2 Mbp) despite different sequencing depths (4.6 and 6.8 Gbp, Table S2).

The lack of correlation between genome recovery and sequencing depth (Fig. 2a) suggests near-saturation of the sequencing effort per SAG. This was further tested by assessing the genome recovery of the two SAGs with largest assemblies using decreasing fractions of the sequenced reads (Fig. 2b). For each subsampled level, the five replicates behaved similarly (SE < 1.5% in both cases) and the dynamics of recovery *vs*. sequencing depth followed a Michaelis-Menten relationship^31^, levelling off at the performed sequencing depth (Fig. 2b). In fact, MAST-4A and MAST-4E SAGs reached about 65% of their genome recovery with only 17% of the reads, and about 80% with half of the reads (Fig. 2b).

From the previous analyses, it appeared that individual SAGs of uncultured protist cells were variable and recovered only a fraction (about 20%) of their genomes, which did not improve by increasing the sequencing effort. This might depend on intrinsic properties of selected cells, their DNA integrity, as well as MDA biases^21^,^28^,^32^. An option to improve genome recovery of uncultured cells would be using partial SAG assemblies to recruit metagenome reads and/or contigs or to serve as training sets for supervised binning efforts of metagenomic data from the same sample. This reassembly of reads has been recently tested on one archaeal SAG of Korarchaeota^33^, resulting in only a slight increase of the genome recovery, from 87% to 89%. Another option is sorting multiple natural cells and performing a targeted metagenomic analysis^34^,^35^,^36^. Thus, the complete chloroplast genome (91 kbp) of *Pelagomonas calceolata* was generated from natural communities^35^. In the case of protists having larger genomes and living in complex communities, sorting natural populations seems less promising, and instead the co-assembly of closely related SAGs that belong to the same species seems a good option. This approach was first used by Rinke and colleagues^37^, which obtained prokaryotic genomes with an estimated completeness of over 90%.

### Determining which SAGs can be co-assembled?

Despite all SAGs from the two MAST lineages had virtually the same 18S rDNA sequence, this could be insufficient to infer genomic homogeneity for co-assembly^38^,^39^, as cells with identical 18S rDNA could be genomically too different^40^. Therefore, we run pairwise comparisons of the SAG sequences using BLASTn (Supplementary Fig. S2). SAGs affiliating to MAST-4A had a slightly lower average nucleotide identity (ANI) among them (95.1% to 99.9%, mean of 97.6 ± 0.8%; Supplementary Fig. S2a) than MAST-4E SAGs (98.4% to 99.6%, mean of 99.1 ± 0.3%; Supplementary Fig. S2b). In each pairwise comparison, and since each SAG contains a different region of the genome (see section below), only a fraction of the assembly could be compared (Supplementary Fig. S2c and S2d). Thus, most SAGs shared less than 50% of their genomic content (average of 32.5% ± 16.2 for MAST-4A and 27.5% ± 9.6 for MAST-4E), except the two replicated SAGs and the pair AA538-E21/AA538-C11, which shared 71.0% and 95.9% respectively. Among MAST-4A, AA538-K07 was atypical, presenting the lowest ANI (95.9% ± 0.4) and the lowest genome overlap with other SAGs (from 3.2% to 8.5%). This SAG presented a second peak in its GC content (data not shown), suggesting the presence of foreign DNA. In the current genomics era, the use of ANI becomes essential to define microbial species. Among prokaryotes, the ANI threshold to adequately define species is above 95–96% in at least 20% of the genome^41^. Similar data on microbial eukaryotic species is not yet available, but a threshold of 97–99% seem to be reasonable based on our results.

**Figure 3:**
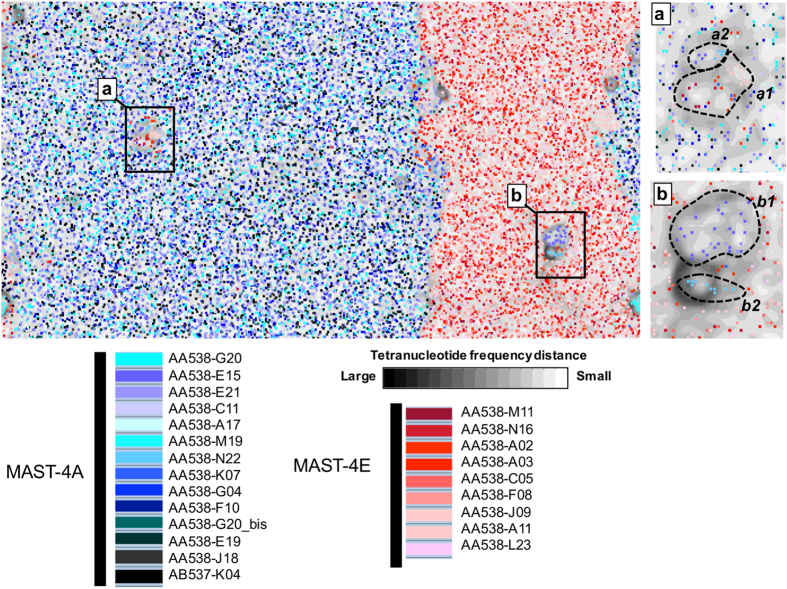
Comparison of tetranucleotide frequencies of SAGs in an ESOM map. Each contig (2.5–5 kbp in size) is represented by a point placed in the map by relatedness and colored according to their provenance from SAGs of MAST-4A (bluish) or MAST-4E (reddish). Note that the map is continuous from top to bottom and side to side. Large differences in tetranucleotide frequencies (black borders) represent natural divisions between taxonomic groups. Two clusters (**a** and **b**) were identified and taxonomically assigned (see text).

Besides the 18S rDNA and ANI comparisons, we also analysed tetranucleotide frequencies coupled to ESOM clustering^42^ to determine if different MAST-4 SAGs have the same genomic features and perhaps identify contigs with deviant signatures. In previous studies, this approach enabled the identification of genomic clusters within prokaryotic assemblages^33^,^43^,^44^. To explore the potential of the ESOM mapping with eukaryotic genomes, we used a selection of published genomes of six photosynthetic and two heterotrophic protists. Fragmented contigs (2.5–5 kbp in size) from each genome formed clear separate clusters (Supplementary Fig. S3). We then adapted this approach to analyse the 23 SAGs used here, represented by 17,029 fragmented contigs (about two thirds from MAST-4A and one third from MAST-4E). As expected, the obtained topography (U-Matrix) representing the structure of the tetranucleotide frequency dataset formed two large clusters that coincided with MAST-4A and MAST-4E contigs (Fig. 3). All SAGs within the same lineage were found in the same region of the map, revealing the same tetranucleotide frequency profile. In addition, we observed two small clusters that contained particular genomic signatures: the subcluster “a” of mitochondrial origin and the subcluster “b” of putative prey origin. A detailed analysis of the contigs within these subclusters identified four subgroups (Fig. 3). The first (a1) included 17 contigs from both lineages related to the mitochondrion of *Cafeteria roenbergensis* (mean similarity of 94%); probably these belonged to MAST-4 mitochondrion. The three other subgroups derived from putative prey DNA. Subcluster a2 contained contigs related to algal mitochondria (2 to *Ostreococcus* spp. and 3 to *Micromonas* spp., with mean similarity of 91% and 98%, respectively), whereas contigs in subclusters b1 and b2 are related to nuclear genome of *Bathycoccus prasinos* (36 contigs with mean similarity of 86%) and *Ostreococcus lucimarinus* (3 contigs with mean similarity of 88%), respectively. The presence of algal DNA in these draft genomes suggests that MAST-4 can ingest picosized algae. Although MAST-4 is generally considered a bacterial grazer, it has been seen eating the picoalgae *Micromonas pusilla* in grazing experiments^45^. Overall, the DNA from algal prey represents a very small fraction of MAST-4 genomes (<0.3% of fragmented contigs).

### Co-assembling individual SAG sequences to by-pass the MDA bias

Based on the tetranucleotide frequency profiles and genomic data (%-GC and ANI values) we decided to co-assemble the Illumina reads of SAGs from the two MAST-4 lineages. The co-assembly of the 14 MAST-4A SAGs yielded 48.1 Mbp and the co-assembly of the 9 MAST-4E SAGs yielded 32.3 Mbp (considering contigs >1 kbp; Table 1). The MAST-4A final co-assembly contained 15,370 contigs with an N50 of 4.5 kbp, while MAST-4E contained 5,679 contigs with an N50 of 10 kbp. The CEGMA analysis searching for 248 core eukaryotic genes identified 184 and 169 orthologs in each genome (Table 1; Supplementary Fig. S4), resulting in an estimated genome completeness of 74.2% and 68.2% in MAST-4A and MAST-4E, respectively. The same analysis done on complete genomes of free-living unicellular eukaryotes sequenced in the standard way (shotgun sequencing from multiple cells of a clonal culture), resulted in only a bit larger recovery estimates, from 78% in *Chlorella variabilis* and *Chlamydomonas reinhardtii* to 96% in *Phytophthora sojae* (Table 1). Overall, the MAST-4 co-assemblies had 4–5 times more conserved proteins and were 5 times longer than individual SAG assemblies.

**Figure 4:**
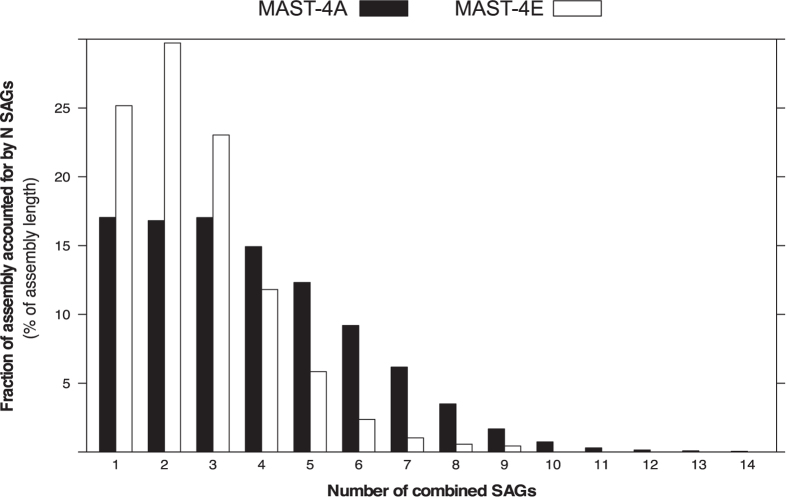
Fractions of the co-assembled genomes of MAST-4A and MAST-4E shared among their respective SAGs (from 1 to 14 cells). The contribution of each SAG was determined through a fragment recruitment analysis of their reads towards the final co-assembly.

As stated before, SAGs from the same lineage had a low sequence overlap, typically around 30% (Supplementary Fig. S2c and S2d), suggesting that each SAG was recovering a different region of the genome. To verify this statement, we mapped the reads of each SAG back to the final co-assembly to determine the contribution of each SAG and the regions of overlap among SAGs. Although a large fraction of the co-assemblies resulted from the combination of several SAGs, a significant part of them, 17% in MAST-4A and 25% in MAST-4E, derived from only one SAG (Fig. 4). More than half of the final co-assembly was obtained with 2–3 SAGs. At the other end, a very small fraction of the final co-assembly (<0.5%) was found in all SAGs (Fig. 4). The latter patterns are likely the result of MDA bias^50^, which seems to randomly amplify a different region of the genome in each SAG.

**Figure 5:**
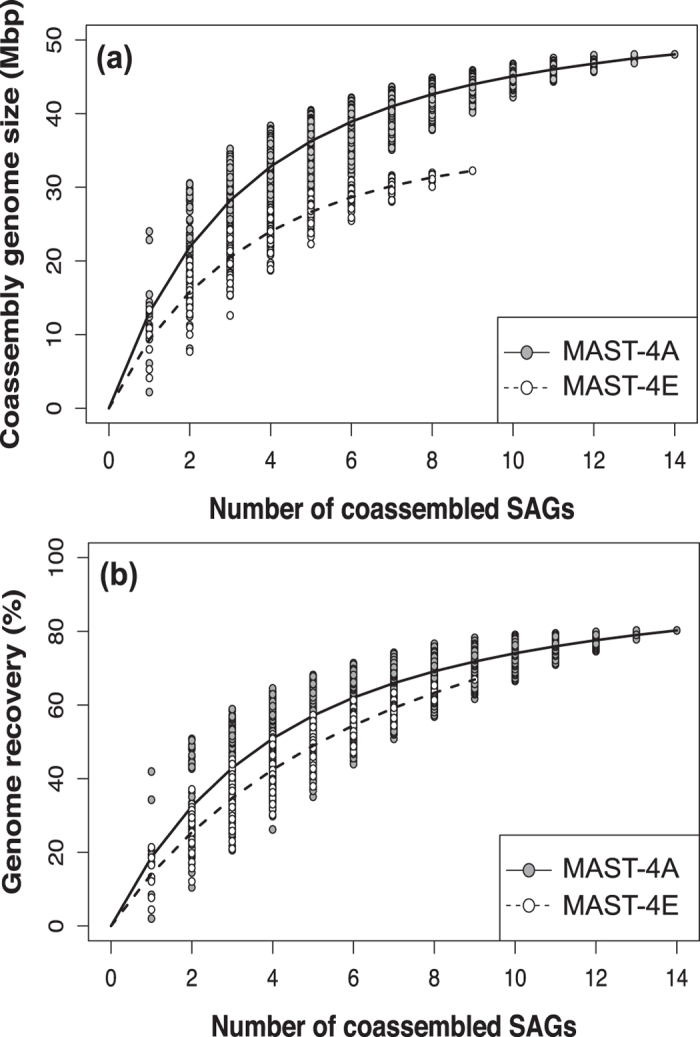
Cumulative genome size (a) and genome recovery (b) calculated when increasing the number of SAGs used for co-assembly.

The relationship between the number of co-assembled SAGs and the size and recovery of the final co-assembly followed a Michaelis-Menten curve^31^ in both cases, with signs of saturation at the highest number of SAGs (Fig. 5). Estimated genome sizes extrapolated from this curve were 62.7 Mbp in MAST-4A and 48.5 Mbp in MAST-4E. These values were similar to those calculated using the genome recovery by CEGMA in the co-assembled genomes, 64.8 and 47.4 Mbp.

### Gain and loss during the co-assembly

Genomic information could be lost during co-assembling if SAGs were not identical, as was the case here. To better understand this potential loss, we compared the contigs of each individual SAG with the final co-assembly using Quast^51^. The vast majority of SAG contigs (>99.5% of their length) matched at >95% identity with the co-assemblies, whereas only a very low proportion of the genomic data present in the individual SAGs was lost, *i*.*e*. 0.3% (±0.2) in MAST-4A and 0.2% (±0.2) in MAST-4E. On the other hand, the final co-assemblies were indeed masking real genetic differences (single nucleotide polymorphisms, indels) of individual cells. On average, 445 (±126) and 122 (±11) mismatches and 125 (±23) and 60 (±7) indels per 100 kbp were detected in MAST-4A and MAST-4E, respectively.

**Figure 6:**
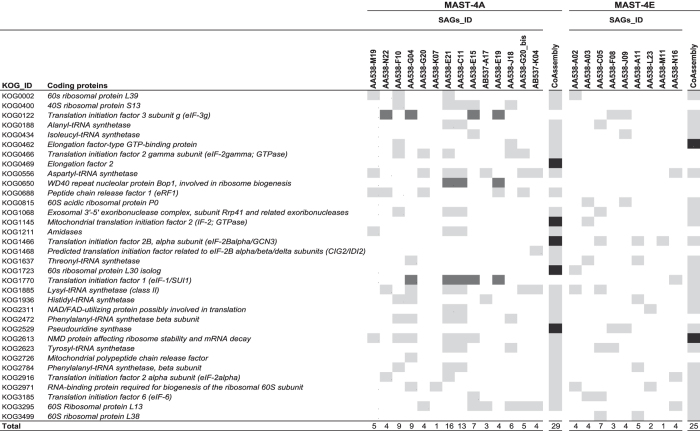
Identification of the 34 CEGs coding for proteins involved in translation, ribosomal structure and biogenesis processes within SAGs and co-assemblies of both lineages. The presence of CEGs among SAGs and co-assembly (light grey) or solely among SAGs (dark grey) or co-assembly (black) are listed here.

Another way to estimate the potential gain and loss of genetic information during the co-assembly is by a detailed analysis of the 248 core eukaryotic genes (CEGs) found within SAGs and the co-assemblies. We illustrated this by focusing on a subset of 34 CEGs coding for proteins involved in translation, ribosomal structure and biogenesis processes (Fig. 6). On the one hand, the final co-assemblies of MAST-4A and MAST-4E retrieved 29 and 25 of these CEGs, whereas each individual SAG retrieved a lower number (1 to 16 in MAST-4A and 1 to 7 in MAST-4E), highlighting the significant gain in retrieved genomic information when co-assembling (Fig. 6). In addition, we also identified several CEGs (5 in MAST-4A and 2 in MAST-4E) found in co-assemblies but absent in individual SAGs, indicating that reads from different SAGs have participated in the assembly of those CEGs. On the other hand, a few CEGs detected in the individual SAGs were not retrieved in their respective co-assembly (KOG1770, KOG0650 and KOG0122 in MAST-4A; Fig. 6). The same analysis done on the complete set of 248 CEGs (Supplementary Fig. S4) revealed 18 and 6 CEGs exclusively found in MAST-4A and MAST-4E co-assemblies, and 33 and 1 CEGs only found in the SAGs (Table 2). These last CEGs were in fact present in the co-assembly but below the detection threshold of CEGMA (at least 70% of the protein length) or in the discarded small contigs (<1 kbp). Overall, we found that all general functions for which conserved genes are indexed were present in both final co-assemblies (Table S3), with only a few exceptions, as the lack of genes involved in the transport and metabolism of inorganic ions in MAST-4E. The retrieval of multiple conserved eukaryotic functions in MAST-4A and MAST-4E supported the adequacy of the co-assembly strategy to increase the amount of retrieved genomic information.

We then compared the sequence of CEGs in the SAGs and in the co-assemblies (Table 2). In most cases, all retrieved sequences were identical (73 of 166 in MAST-4A and 119 of 163 in MAST-4E) or above 95% similarity (53 and 18, respectively). However, there were a few examples of very low sequence identity (for instance in KOG2971 shown in Fig. 6). The reason for these few cases of low similarity was the presence among the SAGs of two CEG variants, very distant among them, and only one was detected in the final co-assembly (since CEGMA only detects one CEG). However, the second variant was also present in the co-assembly. Therefore, even there were two variants of the same CEG coexisting in the population, or the second variant derived from a putative prey. At any rate, we found very high sequence identity among all CEGs retrieved from the SAGs and the co-assemblies.

**Figure 7:**
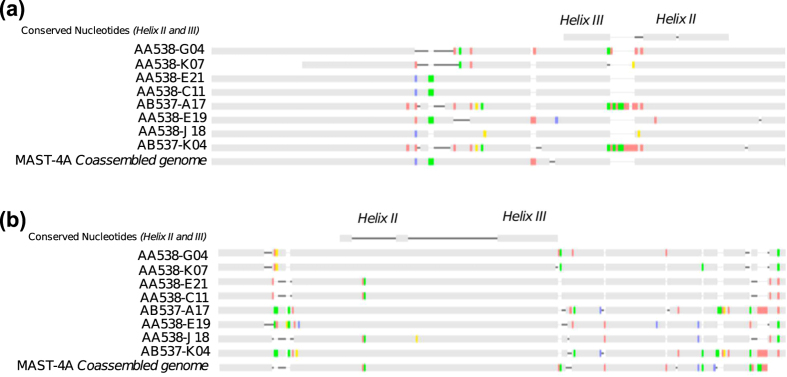
Alignment of the ITS1 (a) and ITS2 (b) regions of individual SAGs and the co-assembly in MAST-4A. Conserved nucleotides in the helices II and III of the two regions were highlighted according to ITS secondary structure models in MAST-4. Differences against a consensus sequence (not shown) are colored as red (A positions), green (T), blue (C), and yellow (G).

We also studied the effect of co-assembling different cells on the universal rDNA operon. We first searched the 18S rDNA sequence and found it in 9 MAST-4A and 6 MAST-4E individual SAGs (Supplementary Fig. S5). Often, the complete rDNA operon was retrieved in a single contig among SAGs, while it was fragmented in the final co-assembly. The rDNA variability was mainly found in the variable Internal Transcribed Spacer (ITS) regions (Supplementary Fig. S5; Fig. 7). Within MAST-4A, the ITS variability correlated with sample origin (Fig. 7), as SAGs from the Indian Ocean (AB537-A17 and AB537-K04) were similar (~97% in both ITS regions) and differed from those in the Mediterranean Sea. Differentiation of MAST-4A populations based on ITS surveys was explained by temperature rather than geographic distance^52^, and our data followed this trend since the samples from the two stations differed in more than 10 °C (Supplementary Table S1). Furthermore, we searched for particular regions in the ITS1 and ITS2 secondary structures (helices II and III) that need to be identical among individuals to be considered from the same biological species^53^. These regions were indeed identical in all SAGs (Fig. 7).

Finally, we predicted genes in the final co-assemblies: 26,676 exons were predicted for MAST-4A and 14,919 for MAST-4E, which resulted in 19,909 and 11,850 predicted proteins (Table 1). A comparable number of proteins, when normalized by the size of its draft genome, were found in MAST-4D, 6,993 genes in the 16.9 Mbp^26^. Compared with published genomes of picosized protists and other heterotrophic flagellates, the number of genes predicted in MAST-4 genomes is relatively high (Table 1), only comparable with the parasitic *Phytophthora* spp, and perhaps some could derive from foreign DNA. On the other hand, the mean gene size (1,657 and 1,723 bp in each lineage) was similar to the values found in other protists genomes (Table 1). Compared to other protists, MAST-4 has compact genomes with few but long introns (Table 1). Finally, a first gene annotation of the two co-assemblies were performed using BLASTp against the KEGG Orthology (KO) database^54^ revealing a total of 2,733 and 2,210 good KO hits for MAST-4A and MAST-4E, respectively (Table 1). Similarly, predicted proteins were also searched against the eukaryotic orthologous groups (KOG) using rpsBLAST and the Conserved Domains and Protein Classification database of NCBI (NCBI-CDD)^55^ and a total of 2,115 and 1,878 KOGs were assigned among the MAST-4A and MAST-4E co-assemblies (Table 1). Nevertheless, since foreign DNA is known to exist in the two co-assemblies, these values are solely indicative and a deeper effort in removing any trace of foreign DNA is still needed to get a better insight of the metabolic machinery of such uncultured organisms. At any rate, our observation of only few contigs coming from putative preys (Fig. 3) suggest a minor impact of foreign DNA in gene prediction and annotation in the two MAST-4 co-assemblies.

## Conclusion

Our study shows that only a fraction (about one fifth) of the genome of a picoeukaryote can be obtained from an individual SAG. SAGs from the same species often retrieve different genome regions, and recovery is hardly improved by increasing the sequencing depth. The co-assembling strategy proposed here has proven its efficiency to bypass these limitations. To ensure the correct mixing of cells from the same species, we established two additional criteria in addition to identical 18S rDNA: a high ANI (>95%) and similar tetranucleotide frequency profiles. By co-assembling SAGs we have access to more genes and functions from uncultured flagellates, although we are also missing intraspecific genetic variability. This strategy can be used and adapted to a range of uncultured protist species, whose genomes would remain unknown or partially known otherwise.

## Methods

### Sample collection and single-cell sorting

Samples for single-cell sorting were collected during the circumglobal *Tara Oceans* expedition^30^ and cryopreserved as described before^22^. Flow cytometry cell sorting, single cell lysis and genomic DNA amplification by Multiple Displacement Amplification (MDA)^56^,^57^ were performed by the Bigelow Laboratory Single Cell Genomics Center (https://scgc.bigelow.org) as previously described^24^,^58^ with a slight modification: 1x SYBR Green I (Life Technologies Corporation) was used instead of Lysotracker Green to stain the cells (Supplementary Fig. S1a). The obtained SAGs were screened by PCR using universal eukaryotic 18S rDNA primers and taxonomically assigned (Supplementary Fig. S1a). A total of 22 SAGs affiliating to the Marine Stramenopiles clade A (MAST-4A) and clade E (MAST-4E) were selected for sequencing. Sample associated environmental metadata are reported in Supplementary Table S1 and more details can be found in PANGAEA^59^.

### SAG sequencing, assembly, quality control and completeness assessment

After purification of the MDA products and generation of 101 bp paired-end libraries, each SAG was sequenced in a 1/8^th^ Illumina HiSeq lane at the Oregon Health & Science University (US) or the National Sequencing Center of Genoscope (France) (Supplementary Table S2 and Supplementary Fig. S1a). Reads were assembled or co-assembled using SPAdes 3.1 or 3.6^60^. In all assemblies, contigs shorter than 1 kbp were discarded. Quality profiles and basic statistics (genome size, number of contigs, N50, GC content) of single SAG assemblies and co-assemblies were generated with Quast^51^. Estimations of genome recovery were done with CEGMA^61^ (Core Eukaryotic Genes Mapping Approach; Supplementary Fig. S1d).

In order to assess if genome completeness in each SAG depended on sequencing effort, reads from the two largest SAG assemblies within each clade were randomly subsampled into 5 different sequencing depths with the seqtk toolkit (https://github.com/lh3/seqtk). Five independent replicates were generated for each sequencing depth using different random number generator seeds. For each pool of subsampled reads, new assemblies and genomes recoveries (contigs >1 kbp) were generated as described above.

### SAG comparisons based on nucleotide identity and tetranucleotide frequency

Nucleotide identity between SAGs was estimated by a pairwise BLAST analysis^62^ between full-length contigs of all SAGs within each clade, with a minimum similarity of 70% and a maximal e-value of 10^−5^. Tetranucleotide frequencies in each individual SAG were calculated using a 1 bp sliding window in both DNA strands in contigs between 2.5 and 5 kbp in size with a custom Perl script^43^ and clustered using ESOM^42^ (Emergent Self-organizing Maps; Supplementary Fig. S1b). Raw data were normalized using robust estimates of mean and variance (“Robust ZT” option) and trained according to Dick and colleagues^43^ with the k-Batch algorithm and Euclidean grid distance. Sub-clusters of interest were isolated to identify the corresponding contigs by a BLASTn analysis against the NCBI-nt and NCBI-RefSeq (including organelles genomes) databases^63^. Blast hits (similarity > 80%, e-value < 10^−5^) were taxonomically assigned.

### Genome analysis using fragment recruitment tools

Original reads were mapped back to their corresponding co-assembly using bowtie2 with default parameters^64^ (Supplementary Fig. S1c). The reads alignments (BAM file) obtained were processed using samtools^65^, BEDTools^66^, QualiMap2^67^ and custom perl scripts. Then a comparison of each individual SAG assembly against the co-assembly as a reference for both MASTs was performed using Quast^51^.

### Analysis of Core Eukaryotic Genes (CEGs) and of the rDNA operon

A subset of 248 universal CEGs within each SAG and the two final co-assemblies were identified with CEGMA^61^. For each detected CEG, amino acids sequences were aligned using Clustal-Omega^68^. These alignments were then used to calculate distance matrices based on percent identities for each sequence pair.

We searched for contigs containing the 18S rDNA sequence in all individual MAST-4 SAGs and the co-assembly. The complete rDNA operon sequences were aligned using ClustalW^69^, as implemented in the Geneious package^70^. Internal Transcribed Spacer regions (ITS1 and ITS2) were identified and annotated based on a previous work on ITS secondary structures of MAST-4^53^.

### Gene prediction of co-assembled genomes and taxonomic profiling

The initial set of CEGs predicted with CEGMA were used to train the Augustus *ab initio* gene predictor^71^ prior to its execution on the full co-assembly using defaults parameters (Supplementary Fig. S1d). Genes were annotated using BLASTp (e-value < 10^−5^) and rpsBLAST against, respectively, KEGG Orthology^54^ and the Conserved Domains and Protein Classification database of NCBI (NCBI-CDD)^55^. BLASTp hits with at least 100 bp alignments including at least 30% of query coverage and >25% similarity were kept.

## Additional Information

**Accession codes**: Sequence data is available at ENA (http://www.ebi.ac.uk/services/tara-oceans-data) with accession codes ERR1138643-ERR1138646, ERR1189843-ERR1189844, ERR1189847, ERR1198925, ERR1198927-ERR1198928, ERR1198936, ERR1198938, ERR1198941, ERR1198946, ERR1198948-ERR1198950, ERR1198954 and ERR1744377- ERR1744380. Co-assemblies are available on request.

**How to cite this article:** Mangot, J.-F. *et al*. Accessing the genomic information of unculturable oceanic picoeukaryotes by combining multiple single cells. *Sci. Rep.* **7**, 41498; doi: 10.1038/srep41498 (2017).

**Publisher's note:** Springer Nature remains neutral with regard to jurisdictional claims in published maps and institutional affiliations.

## Supplementary Material

Supplementary Information

## Figures and Tables

**Table 1:** MAST-4A and MAST-4E assembly properties in comparison to complete published genomes of other small phototrophic and heterotrophic protists.

			Raw assembly size (Mbp)	CEGMA completeness (%)	Number of genes	Mean gene size (bp)	Mean intron density (introns per gene)	Mean intron length (bp)	Number of KOs or GOs*	Number of KOG†	Reference
Stramenopiles	Mar Stram	*MAST*-*4A*	48.1	74.2	19,909	1,657	0.56	260	2,733	2,115	This study
		*MAST*-*4E*	32.3	68.2	11,850	1,723	0.36	332	2,210	1,878	This study
	Bacil	*Thalassiosira pseudonana*	34.5	92.7	11,242	992	1.4		5,473	8,113	46
	Oomyc	*Phytophthora sojae*	95	96.0	19,027	—	—	—	8,714	3,891	19
		*Phytophthora ramorum*	65	95.2	15,743	—	—	—	7,633	3,830	19
Opist	Choan	*Monosiga brevicollis*	42	92.7	9,196	3,004	6.6	174	1,843	3,389	47
Chlorophyta	Mamiell	*Micromonas pusilla* CCMP1545	21.9	83.5	10,575	1,557	0.9	187	4,787	7,086	17
		*Micromonas pusilla* RCC299	20.9	87.1	10,056	1,587	0.57	163	4,911	6,554	17
		*Bathycoccus prasinos*	15	87.5	7,847	—	—	—	3,597	—	18
		*Ostreococcus tauri*	12.5	80.6	8,116	1,257	0.39	187	3,603	5,320	16
	Treb	*Chlorella variabilis*	46.2	77.8	9,791	2,928	—	209	5,372	7,938	48
	Chlor	*Chlamydomonas reinhardtii*	121	77.8	15,143	4,312	0.92	373	6,733	9,435	49

Mar Stram, Marine Stramenopiles. Bacil, Bacillariophyceae. Oomyc, Oomycetes. Opist, Opistokhonta. Choan, Choanoflagellates. Mamiell, Mamiellophyceae. Treb, Trebouxiophyceae. Chlor, Chlorophyceae.

Assembly features of MAST-4A and MAST-4E have been calculated on contigs longer than 1 kb.

Assembly features of published genomes were retrieved from their respective publications or, when missed, from the JGI genome portal (http://genome.jgi.doe.gov). Additionally, their CEGMA completeness (contigs > 1 kb) were also calculated here.

Missing data are shown by the symbol (—).

^*^KOs, KEGG Orthology. GOs, Gene Ontology.

^†^KOGs, Eukaryotic Orthologous Groups.

**Table 2:** Summary of the 248 CEGMA eukaryotic core genes (CEGs) determined in SAGs and co-assemblies of both MAST lineages.

Lineage	Number of CEGs detected						
	In SAGs and Co-assembly						
	*100*%*	≧*95*%	<*95*%	*NA*☥	Total	Solely in SAGs	Solely in Co-assembly
MAST-4A	*73*	*53*	*29*	*11*	166	33	18
MAST-4E	*119*	*18*	*15*	*11*	163	1	6

^*^Mean amino acid sequence identity of CEGs found in several SAGs.

^☥^NA: Not applicable, since these CEGs are found in only one SAG.
