# Supplementary material for: Accessing the genomic information of unculturable oceanic picoeukaryotes by combining multiple single cells
Source: Sci Rep. 2017 Jan 27;7:41498. doi: 10.1038/srep41498 (PMC5269757; doi:10.1038/srep41498)
Supplement: Supplementary Information [file srep41498-s1.pdf]

Supplementary information

Accessing the genomic information of unculturable oceanic picoeukaryotes by combining multiple single cells

Jean-François Mangot[1*], Ramiro Logares[1], Pablo Sanchez[1], Fran Latorre[1], Yoann Seeleuthner[2,3,4], Samuel Mondy[2,3,4], Michael E. Sieracki[5,6], Olivier Jaillon[2,3,4], Patrick Wincker[2,3,4], Colomban de Vargas[7,8], Ramon Massana[1*]

Author affiliations

[1] Department of Marine Biology and Oceanography, Institute of Marine Sciences (ICM)–CSIC, Pg. Marítim de la Barceloneta, 37-49, Barcelona E-08003, Spain.

[2]CEA, Institut de Génomique, Génoscope,2 Rue Gaston Crémieux, Evry F-91000, France.

[3] CNRS, UMR 8030, CP5706, Evry, F-91000, France.

[4] Université d'Evry, UMR 8030, CP5706, Evry, F-91000, France.

[5] National Science Foundation, 4201 Wilson Boulevard, Arlington, VA 22230, USA.

[6] Bigelow Laboratory for Ocean Sciences, 60 Bigelow Drive, East Boothbay, ME 04544, USA.

[7] CNRS, UMR 7144, Station Biologique de Roscoff, Place Georges Teissier, Roscoff, F-29680, France.

[8] Sorbonne Universités, UPMC Université Paris 06, UMR 7144, Station Biologique de Roscoff, Place Georges Teissier, Roscoff, F-29680, France.

* Correspondence and requests for materials should be addressed to J-F.M. (jean-francois.mangot@wanadoo.fr) or R.M. (ramonm@icm.csic.es).

Tel: (+34) 93 2309500; Fax: (+34) 93 2309555.

Supplementary Tables

Table S1. Main physical-chemical characteristics of the two sampled stations.

Stations	Coordinates	Sampling date	Depth (m)	Temperature (°C)	Oxygen (µmol kg^{-1})	Salinity (psu)	Chlorophyll (mg $Chl.m^{-3}$)
23	42° 10’ 12" N, 17° 43' 12" E	18/11/2009	55	15.7	224.3	38.4	0.06
41	14° 33' 36" N, 70° 0' 36" E	30/03/2010	58	27.1	148.3	36.5	0.47

Table S2. General sampling and sequencing characteristics of the different individual SAGs of MAST-4A and MAST-4E.

Species	SAGs ID	Stations*	Sequencing depth (Gbp)	Sequencing platforms	CV**
MAST-4A	AA538-M19	23	6.9	Hiseq (Genoscope)	
	AA538-N22	23	8.5	Hiseq (Genoscope)	
	AA538-F10	23	6.0	Hiseq (Genoscope)	
	AA538-G04	23	4.7	Hiseq (Genoscope)	
	AA538-G20†	23	4.6	Hiseq (Genoscope)	
	AA538-K07	23	4.0	Hiseq (Genoscope)	
	AA538-E21	23	5.7	Hiseq (Genoscope)	
	AA538-C11	23	2.7	Hiseq (Oregon)	
	AA538-E15	23	6.4	Hiseq (Genoscope)	
	AB537-A17	41	4.0	Hiseq (Oregon)	
	AA538-E19	23	2.4	Hiseq (Oregon)	
	AA538-G20_bis†	23	6.8	Hiseq (Oregon)	
	AA538-J18	23	4.8	Hiseq (Genoscope)	
	AB537-K04	41	3.5	Hiseq (Oregon)	
MAST-4E	AA538-A02	23	4.5	Hiseq (Genoscope)	
	AA538-A03	23	4.5	Hiseq (Genoscope)	
	AA538-C05	23	4.6	Hiseq (Genoscope)	
	AA538-F08	23	4.0	Hiseq (Genoscope)	
	AA538-J09	23	4.7	Hiseq (Genoscope)	
	AA538-A11	23	6.8	Hiseq (Genoscope)	
	AA538-L23	23	4.4	Hiseq (Genoscope)	
	AA538-M11	23	4.2	Hiseq (Genoscope)	
	AA538-N16	23	4.9	Hiseq (Genoscope)	
Mean (SE)	all MAST-4A SAGs		5.1 (1.7)		34.0%
	all MAST-4E SAGs		4.7 (0.8)		17.3%
	all SAGs		4.9 (1.4)		28.8%

* Stations 23 and 41 are located in the Mediterranean sea (Adriatic Sea) and Indian Ocean (Arabic Sea), respectively (http://taraoceans.sb-roscoff.fr/EukDiv/#figureW1).

** CV: Coefficient of variation = Standard error/mean.

† SAG sequenced by two different sequencing centers, the two sequencing replicates (AA538_G20 and AA538_G20_bis) were kept for further analysis.

Table S3. General functions present in MAST-4A and MAST-4E genomes based on protein classification according to KOGs of the 248 universal CEGMA eukaryotic genes.

Functioning Process	General Functions	Number of COGs expected	Number of COGs in MAST-4A	Number of COGs in MAST-4E
Information storage and processing	Translation, ribosomal structure and biogenesis	34	29	25
	RNA processing and modification	23	18	16
	Transcription	13	6	9
	Replication, recombination and repair	10	9	7
	Chromatin structure and dynamics	0	0	0
	Shared functions	6	5	4
Cellular processes and signalling	Cell cycle control, cell division, chromosome partitioning	2	2	2
	Nuclear structure	0	0	0
	Defence mechanisms	0	0	0
	Signal transduction mechanisms	4	3	3
	Cell wall/membrane/envelope biogenesis	1	0	0
	Cell motility	0	0	0
	Cytoskeleton	3	2	2
	Extracellular structures	0	0	0
	Intracellular trafficking, secretion, and vesicular transport	17	13	13
	Posttranslational modification, protein turnover, chaperones	42	30	31
	Shared functions	6	3	2
Metabolism	Energy production and conversion	22	13	12
	Carbohydrate transport and metabolism	11	8	9
	Amino acid transport and metabolism	2	2	2
	Nucleotide transport and metabolism	6	4	4
	Coenzyme transport and metabolism	2	2	1
	Lipid transport and metabolism	5	3	2
	Inorganic ion transport and metabolism	3	3	0
	Secondary metabolites biosynthesis, transport and catabolism	1	1	1
	Shared functions	4	4	4
Poorly characterized	General function prediction only	19	15	13
	Function unknown	6	4	3
Shared between different functioning process		6	5	4

Supplementary Figures

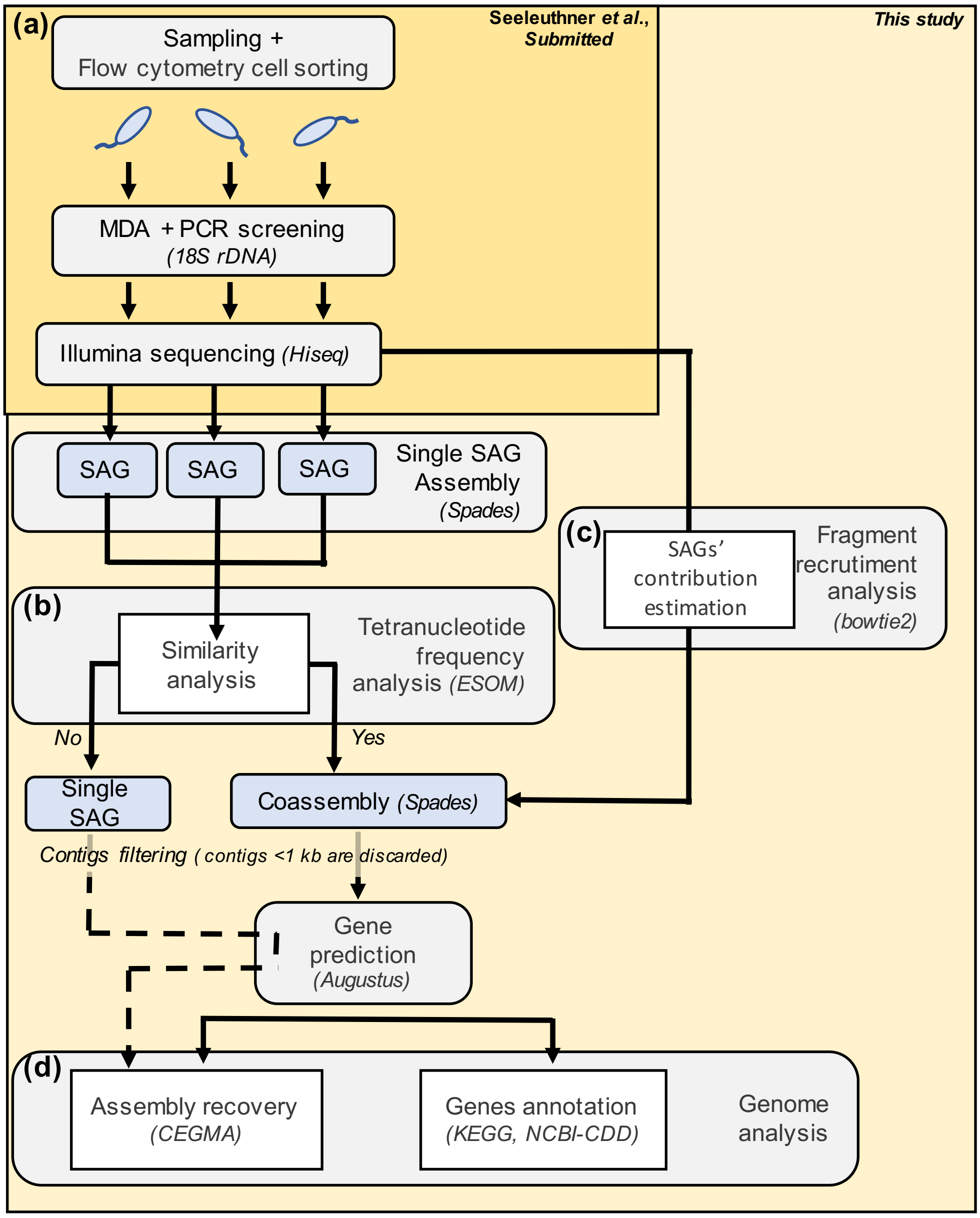

Supplementary Fig. S1. Schematic pipeline of a single-cells co-assembly performed in this study. Details on the sampling, single-cell sorting and SAG sequencing will be available in a concomitant study (Seeleuthner *et al.*, submitted). The rest of the main steps of our co-assembly strategy are described in this paper.

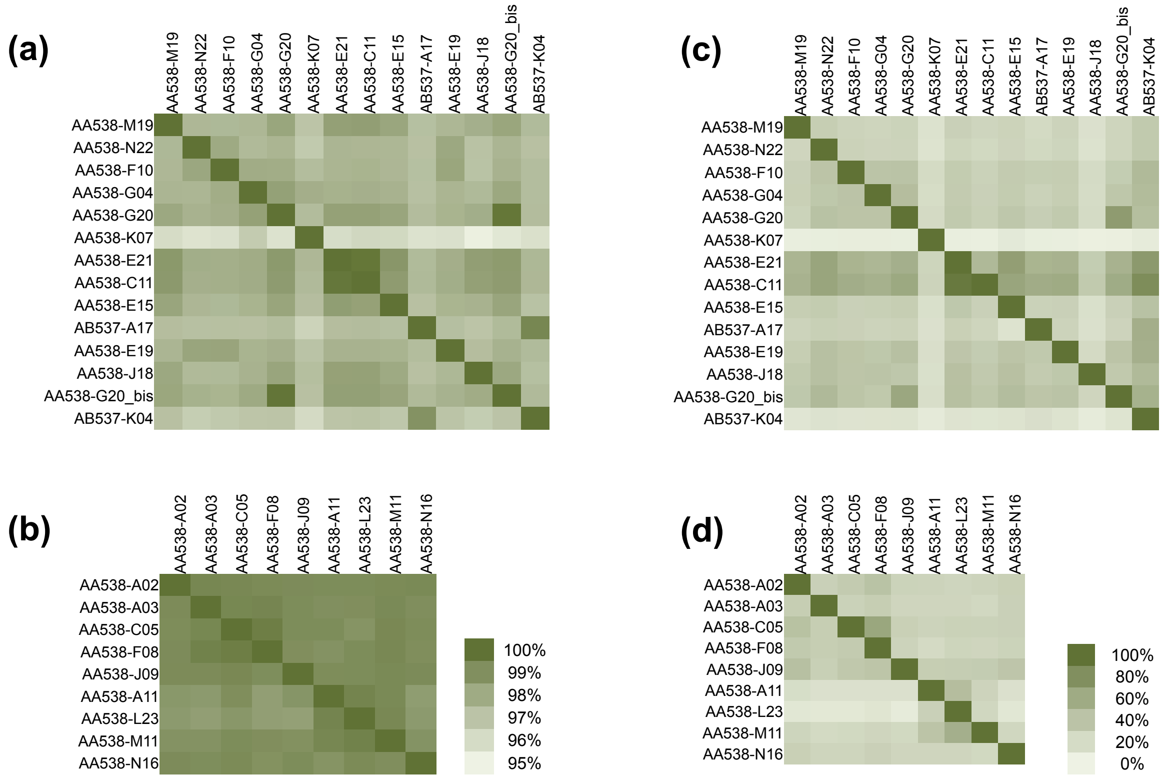

Supplementary Fig. S2. Cross-SAG Blast analysis between MAST-4A and MAST-4E SAGs. Mean pairwise genomic similarity of MAST-4A (**a**) and MAST-4E (**b**) SAGs are represented, together with the percentage of shared nucleotidic regions for MAST-4A (**c**) and MAST-4E (**d**). Values derive from blasting full-length contigs of each SAG against full-length contigs of each sister SAG. Query and subject SAGs are listed in the left and top of each heatmap, respectively.

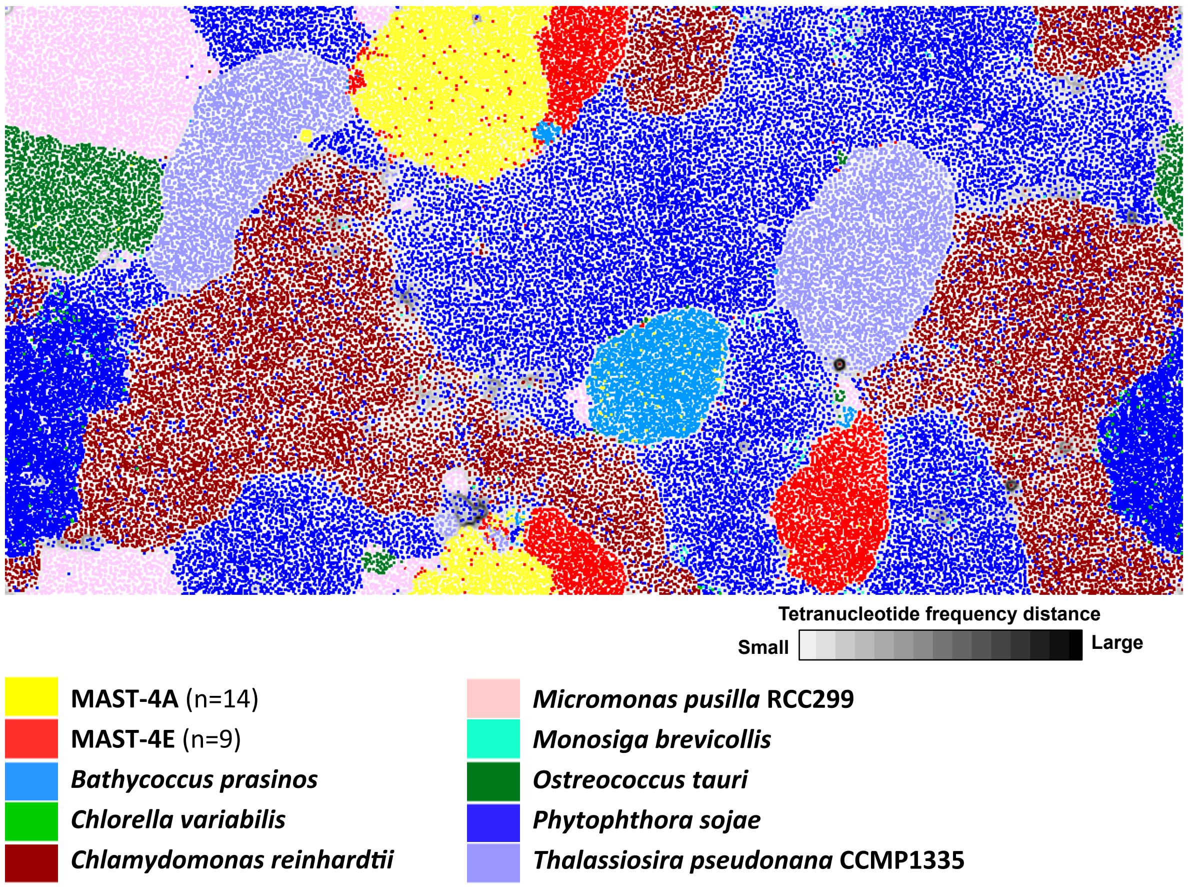

Supplementary Fig. S3. Comparing tetranucleotide frequencies among selected genomes in an ESOM map. Published protist genomes belonging to separate supergroups are combined together with MAST-4A and MAST-4E SAGs. Bestmatches of contigs of 2.5-5 kbp in size are represented by individual points, coloured according to their provenance as MAST-4A (yellow), MAST-4E (red), *Ostreoccocus tauri* (dark green), *Micromonas pusilla* (light blue), *Bathycoccus prasinos* (blue), *Chlorella variabilis* (light green), *Chlamydomonas reinhardtii* (dark red), *Thalassiosira pseudonana* (purple), *Phytophtora sojae* (dark blue) and *Monosiga brevicollis* (pink). Large differences in tetranucleotide frequencies represent natural divisions between taxonomic groups.

KOG_ID	Coding proteins	MAST-4A Mean % identity: SAGs vs SAGs	MAST-4A Mean % identity: SAGs vs Coass.	MAST-4E Mean % identity: SAGs vs SAGs	MAST-4E Mean % identity: SAGs vs Coass.
KOG0002	*60s ribosomal protein L39*	96	98	*NA*	100
KOG0400	*40S ribosomal protein S13*	100	100	*NA*	*NA*
KOG0122	*Translation initiation factor 3 subunit g (eIF-3g)*	100	*NA**	100	100
KOG0188	*Alanyl-tRNA synthetase*	100	100	*NA*	100
KOG0434	*Isoleucyl-tRNA synthetase*	*NA*	100	*NA*	100
KOG0462	*Elongation factor-type GTP-binding protein*	96	98	*NA*	*NA*
KOG0466	*Translation initiation factor 2 gamma subunit (eIF-2gamma; GTPase)*	100	100	*NA*	*NA*
KOG0469	*Elongation factor 2*	*NA*	*NA*	*NA*	*NA*
KOG0556	*Aspartyl-tRNA synthetase*	98	**93**	*NA*	100
KOG0650	*WD40 repeat nucleolar protein Bop1, involved in ribosome biogenesis*	100	*NA*	*NA*	*NA*
KOG0688	*Peptide chain release factor 1 (eRF1)*	99	99	*NA*	*NA*
KOG0815	*60S acidic ribosomal protein P0*	*NA*	*NA*	100	100
KOG1068	*Exosomal 3'-5' exoribonuclease complex, subunit Rrp41 and related exoribonucleases*	Abs[†]	Abs	*NA*	100
KOG1145	*Mitochondrial translation initiation factor 2 (IF-2; GTPase)*	*NA*	*NA*	*NA*	100
KOG1211	*Amidases*	100	97	*NA*	*NA*
KOG1466	*Translation initiation factor 2B, alpha subunit (eIF-2Balpha/GCN3)*	*NA*	*NA*	100	100
KOG1468	*Predicted translation initiation factor related to eIF-2B alpha/beta/delta subunits (CIG2/IDI2)*	Abs	Abs	*NA*	*NA*
KOG1637	*Threonyl-tRNA synthetase*	*NA*	98	**29**	**65**
KOG1723	*60s ribosomal protein L30 isolog*	*NA*	*NA*	*NA*	100
KOG1770	*Translation initiation factor 1 (eIF-1/SUI1)*	100	*NA*	*NA*	100
KOG1885	*Lysyl-tRNA synthetase (class II)*	95	96	96	98
KOG1936	*Histidyl-tRNA synthetase*	100	99	*NA*	100
KOG2311	*NAD/FAD-utilizing protein possibly involved in translation*	97	97	*NA*	100
KOG2472	*Phenylalanyl-tRNA synthetase beta subunit*	100	99	*NA*	*NA*
KOG2529	*Pseudouridine synthase*	*NA*	*NA*	100	100
KOG2613	*NMD protein affecting ribosome stability and mRNA decay*	95	95	*NA*	*NA*
KOG2623	*Tyrosyl-tRNA synthetase*	97	98	100	100
KOG2726	*Mitochondrial polypeptide chain release factor*	*NA*	100	*NA*	*NA*
KOG2784	*Phenylalanyl-tRNA synthetase, beta subunit*	100	100	*NA*	100
KOG2916	*Translation initiation factor 2 alpha subunit (eIF-2alpha)*	100	100	*NA*	100
KOG2971	*RNA-binding protein required for biogenesis of the ribosomal 60S subunit*	**59**	**71**	**59**	**79**
KOG3185	*Translation initiation factor 6 (eIF-6)*	*NA*	100	100	100
KOG3295	*60S Ribosomal protein L13*	97	97	*NA*	**89**
KOG3499	*60S ribosomal protein L38*	*NA*	*NA*	100	100
KOG0285	*Pleiotropic regulator 1*	*NA*	*NA*	100	100
KOG0291	*WD40-repeat-containing subunit of the 18S rRNA processing complex*	*NA*	*NA*	*NA*	*NA*
KOG0329	*ATP-dependent RNA helicase*	Abs	*NA*	*NA*	*NA*
KOG0346	*RNA helicase*	97	98	*NA*	*NA*
KOG0820	*Ribosomal RNA adenine dimethylase*	100	*NA*	*NA*	100
KOG0948	*Nuclear exosomal RNA helicase MTR4, DEAD-box superfamily*	100	100	*NA*	*NA*
KOG1137	*mRNA cleavage and polyadenylation factor II complex, BRR5 (CPSF subunit)*	*NA*	96	*NA*	100
KOG1272	*WD40-repeat-containing subunit of the 18S rRNA processing complex*	99	100	100	100
KOG1596	*Fibrillarin and related nucleolar RNA-binding proteins*	*NA*	*NA*	*NA*	**60**
KOG1774	*Small nuclear ribonucleoprotein E*	*NA*	100	100	100
KOG1775	*U6 snRNA-associated Sm-like protein*	100	*NA*	*NA*	100
KOG1780	*Small Nuclear ribonucleoprotein G*	*NA*	*NA*	*NA*	100
KOG1781	*Small Nuclear ribonucleoprotein splicing factor*	99	99	*NA*	*NA*
KOG1795	*U5 snRNP spliceosome subunit*	100	100	100	100
KOG2481	*Protein required for normal rRNA processing*	96	**93**	98	99
KOG4655	*U3 small nucleolar ribonucleoprotein (snoRNP) component*	*NA*	100	100	100
KOG3013	*Exosomal 3'-5' exoribonuclease complex, subunit Rrp4*	*NA*	*NA*	*NA*	*NA*
KOG3167	*Box H/ACA snoRNP component, involved in ribosomal RNA pseudouridinylation*	99	*NA*	99	100
KOG3448	*Predicted snRNP core protein*	100	100	100	100
KOG3459	*Small nuclear ribonucleoprotein (snRNP) Sm core protein*	100	100	100	100
KOG3482	*Small nuclear ribonucleoprotein (snRNP) SMF*	100	100	Abs	Abs
KOG3503	*H/ACA snoRNP complex, subunit NOP10*	100	100	100	100
KOG2781	*U3 small nucleolar ribonucleoprotein (snoRNP) component*	*NA*	*NA*	*NA*	*NA*
KOG0261	*RNA polymerase III, large subunit*	99	99	100	100
KOG0871	*Class 2 transcription repressor NC2, beta subunit (Dr1)*	*NA*	*NA*	*NA*	*NA*
KOG1534	*Putative transcription factor FET5*	*NA*	**89**	*NA*	100
KOG1597	*Transcription initiation factor TFIIB*	*NA*	*NA*	*NA*	*NA*
KOG2680	*DNA helicase TIP49, TBP-interacting protein*	**92**	95	*NA*	**91**
KOG2906	*RNA polymerase III subunit C11*	97	98	100	100
KOG3218	*RNA polymerase, 25-kDa subunit (common to polymerases I, II and III)*	99	100	100	100
KOG3297	*DNA-directed RNA polymerase subunit E'*	*NA*	*NA*	*NA*	*NA*
KOG3400	*RNA polymerase subunit 8*	99	*NA*	*NA*	100
KOG3404	*G10 protein/predicted nuclear transcription regulator*	*NA*	*NA*	97	98
KOG3405	*RNA polymerase subunit K*	*NA*	*NA*	*NA*	*NA*
KOG3497	*DNA-directed RNA polymerase, subunit RPB10*	*NA*	100	100	100
KOG4392	*RNA polymerase, subunit L*	*NA*	*NA*	97	98
KOG0477	*DNA replication licensing factor, MCM2 component*	96	**93**	*NA*	97
KOG0481	*DNA replication licensing factor, MCM5 component*	Abs	*NA*	Abs	100
KOG0969	*DNA polymerase delta, catalytic subunit*	98	99	*NA*	97
KOG0989	*Replication factor C, subunit RFC4*	**92**	96	*NA*	100
KOG0991	*Replication factor C, subunit RFC2*	99	99	*NA*	*NA*
KOG1532	*GTPase XAB1, interacts with DNA repair protein XPA*	*NA*	100	*NA*	*NA*
KOG1636	*DNA polymerase delta processivity factor (proliferating cell nuclear antigen)*	100	100	*NA*	*NA*
KOG1942	*DNA helicase, TBP-interacting protein*	100	100	100	100
KOG2519	*5'-3' exonuclease*	*NA*	100	*NA*	100
KOG2851	*Eukaryotic-type DNA primase, catalytic (small) subunit*	98	*NA*	*NA*	100
KOG2572	*Ribosome biogenesis protein - Nop58p/Nop5p*	97	**94**	*NA*	97
KOG3387	*60S ribosomal protein 15.5kD/SNU13, NHP2/L7A family (includes ribonuclease P subunit p38)*	98	*NA*	100	100
KOG2535	*RNA polymerase II elongator complex, subunit ELP3/histone acetyltransferase*	Abs	Abs	Abs	Abs
KOG1123	*RNA polymerase II transcription initiation/nucleotide excision repair factor TFIIH, 3'-5' helicase subunit SSL2*	Abs	Abs	*NA*	*NA*
KOG2807	*RNA polymerase II transcription initiation/nucleotide excision repair factor TFIIH, subunit SSL1*	99	99	*NA*	*NA*
KOG2044	*5'-3' exonuclease HKE1/RAT1*	100	97	**89**	96
KOG0018	*Structural maintenance of chromosome protein 1 (sister chromatid cohesion complex Cohesin, subunit SMC1)*	*NA*	*NA*	*NA*	99
KOG0964	*Structural maintenance of chromosome protein 3 (sister chromatid cohesion complex Cohesin, subunit SMC3)*	98	98	100	100
KOG0211	*Protein phosphatase 2A regulatory subunit A and related proteins*	95	97	*NA*	98
KOG0279	*G protein beta subunit-like protein*	**56**	**74**	100	100
KOG3205	*Rho GDP-dissociation inhibitor*	*NA*	*NA*	*NA*	*NA*
KOG3275	*Zinc-binding protein of the histidine triad (HIT) family*	99	99	**48**	**48**
KOG1322	*GDP-mannose pyrophosphorylase/mannose-1-phosphate guanylyltransferase*	*NA*	*NA*	*NA*	*NA*
KOG1374	*Gamma tubulin*	100	**94**	*NA*	100
KOG1523	*Actin-related protein Arp2/3 complex, subunit ARPC1/p41-ARC*	100	*NA*	*NA*	99
KOG3174	*F-actin capping protein, beta subunit*	100	100	*NA*	*NA*
KOG0077	*Vesicle coat complex COPII, GTPase subunit SAR1*	*NA*	**92**	Abs	Abs
KOG0276	*Vesicle coat complex COPI, beta' subunit*	98	99	*NA*	*NA*
KOG0292	*Vesicle coat complex COPI, alpha subunit*	100	100	*NA*	100
KOG0780	*Signal recognition particle, subunit Srp54*	100	97	*NA*	*NA*
KOG0861	*SNARE protein YKT6, synaptobrevin/VAMP syperfamily*	*NA*	**82**	100	100
KOG0862	*Synaptobrevin/VAMP-like protein SEC22*	*NA*	*NA*	100	100
KOG0937	*Adaptor complexes medium subunit family*	99	100	*NA*	100
KOG0985	*Vesicle coat protein clathrin, heavy chain*	*NA*	*NA*	*NA*	*NA*
KOG1058	*Vesicle coat complex COPI, beta subunit*	98	99	*NA*	*NA*
KOG1299	*Vacuolar sorting protein VPS45/Stt10 (Sec1 family)*	**23**	**61**	*NA*	95
KOG1688	*Golgi proteins involved in ER retention (RER)*	*NA*	100	100	100
KOG1733	*Mitochondrial import inner membrane translocase, subunit TIM13*	*NA*	*NA*	*NA*	*NA*
KOG2104	*Nuclear transport factor 2*	*NA*	100	100	100
KOG3232	*Vacuolar assembly/sorting protein DID2*	**60**	Abs	*NA*	100
KOG3330	*Transport protein particle (TRAPP) complex subunit*	*NA*	*NA*	Abs	Abs
KOG3343	*Vesicle coat complex COPI, zeta subunit*	*NA*	100	100	100
KOG3479	*Mitochondrial import inner membrane translocase, subunit TIM9*	*NA*	*NA*	*NA*	100
KOG0174	*20S proteasome, regulatory subunit beta type PSMB6/PSMB9/PRE3*	100	Abs	Abs	Abs
KOG0175	*20S proteasome, regulatory subunit beta type PSMB5/PSMB8/PRE2*	*NA*	*NA*	99	99
KOG0176	*20S proteasome, regulatory subunit alpha type PSMA5/PUP2*	*NA*	*NA*	*NA*	*NA*
KOG0177	*20S proteasome, regulatory subunit beta type PSMB2/PRE1*	100	100	*NA*	100
KOG0179	*20S proteasome, regulatory subunit beta type PSMB1/PRE7*	100	*NA*	*NA*	*NA*
KOG0180	*20S proteasome, regulatory subunit beta type PSMB3/PUP3*	*NA*	100	100	100
KOG0181	*20S proteasome, regulatory subunit alpha type PSMA2/PRE8*	100	100	100	100
KOG0182	*20S proteasome, regulatory subunit alpha type PSMA6/SCL1*	100	100	100	100
KOG0184	*20S proteasome, regulatory subunit alpha type PSMA3/PRE10*	**93**	**92**	100	100
KOG0357	*Chaperonin complex component, TCP-1 epsilon subunit (CCT5)*	*NA*	100	100	100
KOG0358	*Chaperonin complex component, TCP-1 delta subunit (CCT4)*	*NA*	*NA*	*NA*	100
KOG0359	*Chaperonin complex component, TCP-1 zeta subunit (CCT6)*	100	100	100	100
KOG0361	*Chaperonin complex component, TCP-1 eta subunit (CCT7)*	98	99	*NA*	100
KOG0363	*Chaperonin complex component, TCP-1 beta subunit (CCT2)*	*NA*	100	99	100
KOG0364	*Chaperonin complex component, TCP-1 gamma subunit (CCT3)*	*NA*	100	*NA*	*NA*
KOG0365	*Beta subunit of farnesyltransferase*	98	99	100	100

KOG0366	Protein geranylgeranyltransferase type II, beta subunit			NA	NA			NA	100
KOG0419	Ubiquitin-protein ligase			NA	96			NA	100
KOG0424	Ubiquitin-protein ligase			**62**	**71**			100	100
KOG0687	26S proteasome regulatory complex, subunit RPN7/PSMD6			100	100			NA	NA
KOG0729	26S proteasome regulatory complex, ATPase RPT1			100	99			NA	100
KOG0741	AAA+-type ATPase			100	100			NA	NA
KOG0894	Ubiquitin-protein ligase			97	NA			NA	NA
KOG0960	Mitochondrial processing peptidase, beta subunit, and related enzymes (insulinase superfamily)			100	100			100	100
KOG1349	Gpi-anchor transamidase			100	100			100	100
KOG1358	Serine palmitoyltransferase			NA	NA			NA	100
KOG1439	RAB proteins geranylgeranyltransferase component A (RAB escort protein)			100	99			99	99
KOG1463	26S proteasome regulatory complex, subunit RPN6/PSMD11			98	99			NA	NA
KOG1498	26S proteasome regulatory complex, subunit RPN5/PSMD12			99	100			100	100
KOG1555	26S proteasome regulatory complex, subunit RPN11			100	NA			NA	NA
KOG1556	26S proteasome regulatory complex, subunit RPN8/PSMD7			Abs	96			100	100
KOG1760	Molecular chaperone Prefoldin, subunit 4			98	NA			100	100
KOG1769	Ubiquitin-like proteins			**70**	**62**			**52**	**76**
KOG1816	Ubiquitin fusion-degradation protein			NA	NA			NA	NA
KOG1872	Ubiquitin-specific protease			99	99			NA	NA
KOG2004	Mitochondrial ATP-dependent protease PIM1/LON			97	100			100	100
KOG2707	Predicted metalloprotease with chaperone activity (RNAse H/HSP70 fold)			**88**	NA			100	100
KOG2908	26S proteasome regulatory complex, subunit RPN9/PSMD13			NA	98			NA	100
KOG2930	SCF ubiquitin ligase, Rbx1 component			NA	99			100	100
KOG3048	Molecular chaperone Prefoldin, subunit 5			98	NA			100	100
KOG3493	Ubiquitin-like protein			NA	NA			NA	NA
KOG3313	Molecular chaperone Prefoldin, subunit 3			100	NA			100	100
KOG1746	Defender against cell death protein/oligosaccharyltransferase, epsilon subunit			NA	NA			Abs	Abs
KOG2606	OTU (ovarian tumor)-like cysteine protease			NA	NA			100	100
KOG1373	Transport protein Sec61, alpha subunit			**68**	**84**			NA	NA
KOG1241	Karyopherin (importin) beta 1			Abs	Abs			NA	NA
KOG1727	Microtubule-binding protein (translationally controlled tumor protein)			NA	NA			NA	NA
KOG3285	Spindle assembly checkpoint protein			98	99			NA	NA
KOG0225	Pyruvate dehydrogenase E1, alpha subunit			98	99			100	100
KOG0233	Vacuolar H+-ATPase V0 sector, subunit c''			NA	100			100	100
KOG0524	Pyruvate dehydrogenase E1, beta subunit			99	100			NA	NA
KOG0559	Dihydrolipoamide succinyltransferase (2-oxoglutarate dehydrogenase, E2 subunit)			NA	NA			NA	98
KOG1159	NADP-dependent flavoprotein reductase			NA	100			NA	100
KOG1335	Dihydrolipoamide dehydrogenase			NA	99			NA	NA
KOG1350	F0F1-type ATP synthase, beta subunit			**82**	**73**			NA	NA
KOG1353	F0F1-type ATP synthase, alpha subunit			**60**	**66**			**71**	**57**
KOG1647	Vacuolar H+-ATPase V1 sector, subunit D			98	NA			Abs	Abs
KOG1662	Mitochondrial F1F0-ATP synthase, subunit OSCP/ATP5			100	100			NA	100
KOG1664	Vacuolar H+-ATPase V1 sector, subunit E			Abs	NA			NA	NA
KOG1758	Mitochondrial F1F0-ATP synthase, subunit delta/ATP16			NA	100			NA	NA
KOG2415	Electron transfer flavoprotein ubiquinone oxidoreductase			NA	NA			NA	100
KOG2451	Aldehyde dehydrogenase			99	100			99	100
KOG2792	Putative cytochrome C oxidase assembly protein			NA	NA			100	100
KOG2909	Vacuolar H+-ATPase V1 sector, subunit C			Abs	NA			Abs	Abs
KOG3049	Succinate dehydrogenase, Fe-S protein subunit			99	NA			NA	NA
KOG3052	Cytochrome c1			NA	NA			NA	NA
KOG3180	Electron transfer flavoprotein, beta subunit			100	NA			NA	NA
KOG3361	Iron binding protein involved in Fe-S cluster formation			98	NA			NA	100
KOG3432	Vacuolar H+-ATPase V1 sector, subunit F			100	100			NA	NA
KOG3954	Electron transfer flavoprotein, alpha subunit			NA	NA			NA	NA
KOG0563	Glucose-6-phosphate 1-dehydrogenase			**80**	**76**			100	100
KOG1367	3-phosphoglycerate kinase			**69**	**83**			**58**	**59**
KOG1458	Fructose-1,6-bisphosphatase			100	100			100	100
KOG1643	Triosephosphate isomerase			**58**	NA			NA	100
KOG2446	Glucose-6-phosphate isomerase			NA	NA			NA	NA
KOG2531	Sugar (pentulose and hexulose) kinases			NA	NA			NA	**91**
KOG2537	Phosphoglucomutase/phosphomannomutase			99	99			NA	100
KOG2638	UDP-glucose pyrophosphorylase			NA	100			NA	100
KOG2653	6-phosphogluconate dehydrogenase			**37**	**69**			NA	100
KOG2757	Mannose-6-phosphate isomerase			NA	NA			NA	NA
KOG3147	6-phosphogluconolactonase - like protein			100	97			NA	NA
KOG1549	Cysteine desulfurase NFS1			100	100			Abs	100
KOG2770	Aminomethyl transferase			NA	NA			100	100
KOG0888	Nucleoside diphosphate kinase			**52**	**76**			NA	100
KOG1112	Ribonucleotide reductase, alpha subunit			97	99			NA	100
KOG1355	Adenylosuccinate synthase			98	99			NA	100
KOG1712	Adenine phosphoribosyl transferases			99	NA			100	100
KOG1800	Ferredoxin/adrenodoxin reductase			NA	NA			NA	NA
KOG3222	Inosine triphosphate pyrophosphatase			100	100			NA	NA
KOG1540	Ubiquinone biosynthesis methyltransferase COQ5			NA	98			NA	100
KOG2017	Molybdopterin synthase sulfurylase			97	97			NA	NA
KOG1390	Acetyl-CoA acetyltransferase			99	99			NA	NA
KOG1393	Hydroxymethylglutaryl-CoA synthase			NA	100			NA	96
KOG1889	Putative phosphoinositide phosphatase			NA	NA			NA	NA
KOG2833	Mevalonate pyrophosphate decarboxylase			100	100			NA	NA
KOG3189	Phosphomannomutase			99	NA			NA	NA
KOG0209	P-type ATPase			Abs	Abs			NA	NA
KOG0876	Manganese superoxide dismutase			NA	100			NA	NA
KOG2825	Putative arsenite-translocating ATPase			NA	99			NA	NA
KOG0142	Isopentenyl pyrophosphate:dimethylallyl pyrophosphate isomerase			**65**	**78**			**62**	**81**
KOG1185	Thiamine pyrophosphate-requiring enzyme			**70**	**63**			NA	100
KOG1394	3-oxoacyl-(acyl-carrier-protein) synthase (I and II)			100	100			NA	NA
KOG2575	Glucosyltransferase - Alg6p			NA	NA			NA	100
KOG3855	Monooxygenase involved in coenzyme Q (ubiquinone) biosynthesis			100	100			NA	**80**
KOG0302	Ribosome Assembly protein			98	NA			NA	100
KOG0376	Serine-threonine phosphatase 2A, catalytic subunit			100	100			NA	NA
KOG0567	HEAT repeat-containing protein			100	100			100	100
KOG0927	Predicted transporter (ABC superfamily)			99	100			100	100
KOG1235	Predicted unusual protein kinase			100	100			NA	NA
KOG1533	Predicted GTPase			NA	NA			**51**	**75**
KOG1535	Predicted fumarylacetoacetate hydralase			**73**	**87**			**25**	**63**
KOG1541	Predicted protein carboxyl methylase			100	100			100	100
KOG2036	Predicted P-loop ATPase fused to an acetyltransferase			96	95			NA	NA
KOG2703	C4-type Zn-finger protein			98	NA			NA	NA
KOG2719	Metalloprotease			100	100			Abs	Abs
KOG2728	Uncharacterized conserved protein with similarity to phosphopantothenoylcysteine synthetase/decarboxylase			98	99			NA	NA
KOG2775	Metallopeptidase			99	100			NA	NA
KOG2785	C2H2-type Zn-finger protein			NA	100			NA	NA
KOG2948	Predicted metal-binding protein			98	**93**			100	100
KOG3157	Proline synthetase co-transcribed protein			100	100			100	100
KOG3163	Uncharacterized conserved protein related to ribosomal protein S8E			97	**90**			NA	100
KOG3239	Density-regulated protein related to translation initiation factor 1 (eIF-1/SUI1)			Abs	NA			NA	100
KOG3349	Predicted glycosyltransferase			98	NA			NA	100
KOG0271	Notchless-like WD40 repeat-containing protein			Abs	100			NA	NA
KOG1088	Uncharacterized conserved protein			100	100			NA	NA
KOG1980	Uncharacterized conserved protein			NA	100			NA	100
KOG2967	Uncharacterized conserved protein			NA	NA			99	99
KOG3237	Uncharacterized conserved protein			NA	100			NA	100
KOG3318	Predicted membrane protein			NA	NA			NA	NA
KOG2303	Predicted NAD synthase, contains CN hydrolase domain			**65**	**65**			NA	100
KOG2035	Replication factor C, subunit RFC3			100	100			NA	NA
KOG1099	SAM-dependent methyltransferase/cell division protein FtsJ			**93**	96			NA	NA
KOG2874	rRNA processing protein			**90**	**89**			99	99
KOG0025	Zn2+-binding dehydrogenase (nuclear receptor binding factor-1)			NA	NA			NA	NA
KOG0062	ATPase component of ABC transporters with duplicated ATPase domains/Translation elongation factor EF-3b			**63**	**53**			**33**	**66**
	**Total**	51 42 46 42 41 5 104 85 48 47 43 21 42 31	184			41 53 41 33 41 46 19 11 30	169		

in bold, mean identity < 95%

* *NA* : Not applicable

† Abs : protein sequences of the predicted CEGs were not retrieved in the file generated by CEGMA.

Supplementary Fig. S4. Identification of the 248 CEGs within SAGs and co-assemblies of both lineages. The presence of CEGs among SAGs and co-assembly (light grey) or solely among SAGs (dark grey) or co-assembly (black) are here listed. The mean amino acid

identities of the retrieved CEGs were calculated among SAGs ("SAGs vs SAGs") and between SAGs and co-assembly ("SAGs vs Coass.").

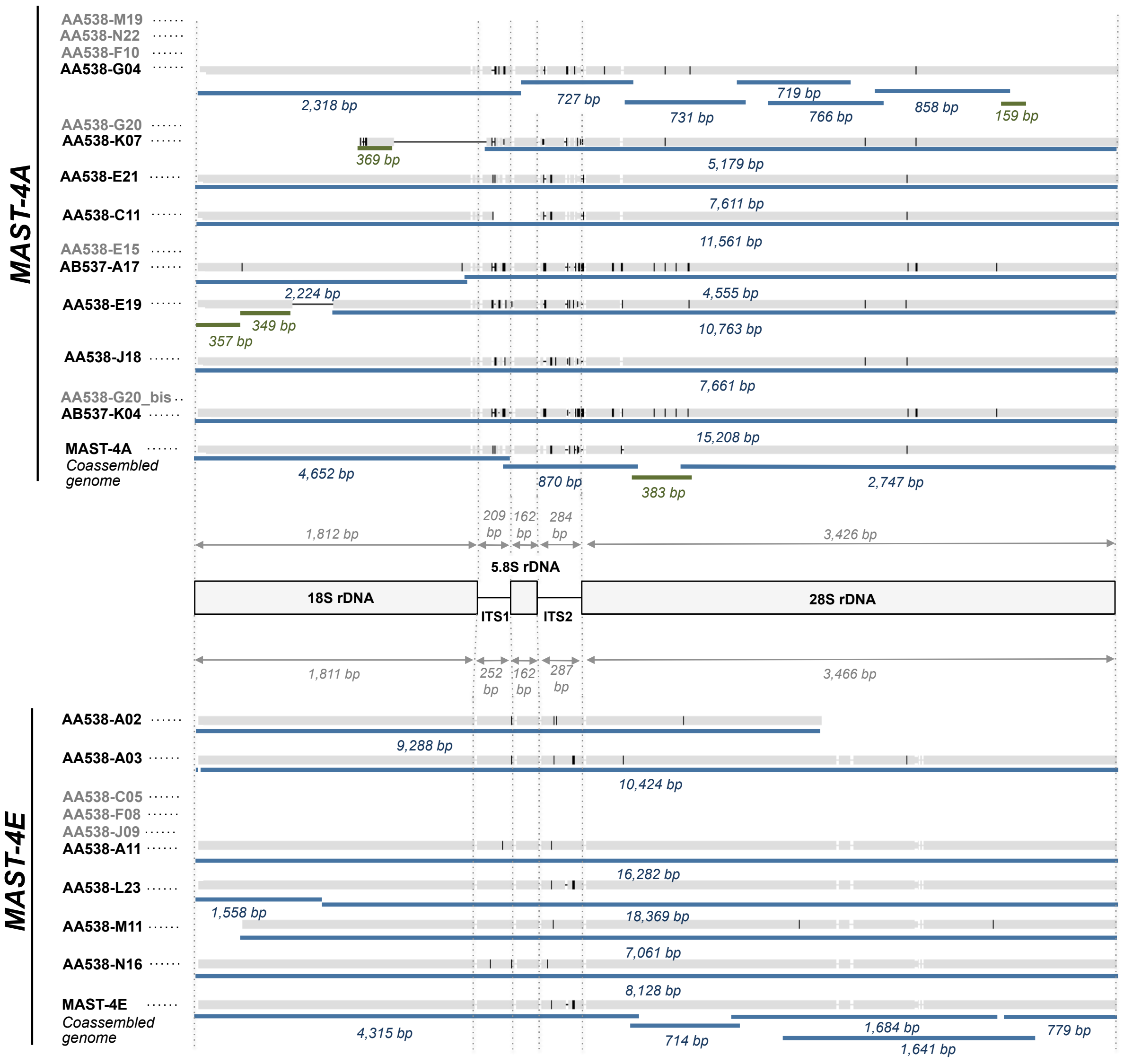

Supplementary Fig. S5. Retrieval of the rDNA operon in SAGs of the two MAST-4 lineages. Sequences of MAST-4A (top) and MAST-4E (bottom) individual SAGs containing the rDNA operon were aligned with their corresponding co-assembled genomes. SAGs without rDNA operon contigs are shown in grey. The position and length of contigs from each SAG and co-assemblies necessary to reconstruct the rDNA operon are shown (contigs <500 bp in green and >500 bp in blue). Differences in individual SAGs against the consensus sequence are marked.
